# An Interesting Case of Barbiturate Automatism and Review of Literature

**DOI:** 10.1155/2013/713065

**Published:** 2013-10-13

**Authors:** Sankalp Gokhale, Ciro Ramos-Estebanez

**Affiliations:** ^1^Department of Neurology, Duke University Hospital, Durham, NC 27710, USA; ^2^Department of Neurology, Neurocritical Care Division, Case Western Reserve University School of Medicine, Cleveland, OH 44106, USA

## Abstract

A 48 year old man with a diagnosis of HIV infection since 1993, on highly active anti-retro viral therapy (HAART) with stable CD4 count and undetectable viral load for years and seizure disorder presented with recurrent drowsiness. His seizures were well controlled on phenobarbitone for years. Repeated laboratory evaluation demonstrated toxic levels of phenobarbitone in his blood. A thorough clinical, psychiatric, laboratory and imaging evaluation did not reveal any obvious etiology for the recurrent barbiturate intoxication in this man. Our findings suggest the possible diagnosis of barbiturate drug automatism in this patient. Though drug automatism is a controversial entity, it merits continued attention. There are recent reports of similar phenomenon with newer sedative agents such as Zolpidem. It is important to be aware of this phenomenon as a possible explanation for recurrent intoxication with barbiturates without a clear etiology for drug overdose.

## 1. Case Report

We report a case of 48-year-old man with a diagnosis of HIV infection since 1993, on HAART (abacavir, lopinavir-ritonavir, and stavudine) with a recent CD4 count of 692, and undetectable viral load for years type II diabetes, and generalized tonic-clonic, complex partial and motor seizures since childhood. His seizures had been well controlled on phenobarbital, levetiracetam, and zonisamide for many years. Prior Electroencephalograms (EEGs) had evidenced right temporal discharges without clinical seizure activity. His brain magnetic resonance imaging had remained normal. He resides at a group home, where he was responsible for taking his medications. 

The patient presented with repeated admissions due to changes in mental status. He consistently became somnolent, arousable to voice, and able to follow simple commands. He had symmetrical fine and course nystagmus. His tone was flaccid and reflexes were diminished with upgoing toes. 

Initially his phenobarbital level was 65.5 mcg/mL (therapeutic level: 15–40 mcg/mL). It increased to 120 mcg/mL during readmission a day after discharge. Once his levels trended down to 23.3 mcg/mL, he was discharged from the hospital and further assistance with medications was initiated at his group home. Several days later, his phenobarbital level was 51.3 mcg/mL. He reported that he was taking phenobarbital at the prescribed doses and denied overdosing himself. He was continued on phenobarbital 120 mg/day. At discharge his phenobarbital level was 41.3 mcg/mL. Hours later, the levels peaked at 112 mcg/mL. Finally, we increased his zonisamide dosing and stopped his phenobarbital. After switching to zonisamide, he did not have recurrent episodes of drowsiness and remained seizure-free.

During this period, he had normal ammonia levels and liver function tests (LFTs). He did not have any systemic infection or metabolic abnormality that could potentially explain high barbiturate levels. Urine toxicology was always exclusively positive for barbiturates. His home's medical staff denied any clinical evidence of chronic drug intoxication. He underwent 24 hour EEG monitoring which did not show evidence of seizures but showed background encephalopathy. Serial-neuroimaging studies remained negative. There were no changes in medications or his body weight. Indeed, once recovered, his cognition was always intact and at baseline, except for retrograde amnesia limited to the hours leading to an increase of his phenobarbital levels. He consistently denied any dosing mistakes or intentional overdosing. Psychiatric consultation repeatedly ruled out depression and suicidal ideation. Barbiturate addiction was also deemed highly unlikely because the patient never run out the limited amount of pills he received weekly. 

## 2. Discussion

The concept automatism was described as a means to study behavior at lower levels of consciousness [1]. It refers to nonreflexive, complex, and apparently goal-directed behavior performed without full awareness and, therefore, with subsequent partial or complete amnesia [2]. 

Drug automatism has been proposed to occur in the context of a dissociative state [3]. It entails the repeated self-administration of a drug without memory for taking it. It is postulated that after taking certain medications, most notably barbiturates, the individual enters into a different state of consciousness that enables perseveration on the act of redosing till a high degree of sedation has been reached [4]. Uniformly, the subject denies memory of overdosing when he or she recovers from the sedative effects of the inciting medication [5].

Drug automatism has been widely questioned and possibly misrepresented in the recent literature. This situation partly owes to methodological flaws in many reports supporting this idea [5, 6]. Besides, certain confounders such as the medicolegal question of a suicidal attempt [7], the possibility of barbiturate addiction, and medical comorbidities add uncertainty about this entity's bona fide. This situation might lead to overlooking automatism as a source for intoxications. 

We believe that the concept of automatism merits attention. Notably, dissociative states have been described in neurological disease (seizure disorders, parasomnias, and traumatic brain injury), psychiatric conditions (schizophrenia, psychological trauma, and dissociative reactions), intoxications, or withdrawal syndromes. Transient amnestic states have been acknowledged in transient global amnesia, seizures, and in transient psychogenic amnesia [8]. Individuals presenting with transient psychogenic amnesia exhibit normal neuroanatomical studies. However, there is evidence of hypometabolism in the right prefrontal cortex in Fluoro Deoxy Glucose Positron Emission Technology (FDG-PET) studies [9]. Importantly, this area is known to correlate with focal retrograde amnesia for autobiographical events [10]. 

Our case is compelling for it highlights a disconnection between pathophysiological concepts (dissociation and transient psychogenic amnesia) and the clinical acceptance of entities ascribed to them. This report supports the existence of this phenomenon. Indeed, while barbiturates have declined in use, reports of automatism have emerged with other hypnotics, such as Zolpidem. Thereby, and despite the controversial nature of this phenomenon, we feel it merits continued consideration given its significance for patient safety. Besides, a majority of patients requiring these medications have varying degrees of underlying organic brain disease, which makes them particularly vulnerable to the phenomenon of “automatism.” This is particularly relevant as we strive to develop neuromodulatory medications for treatment of disorders ranging from autism to seizures.
